# Neonatal outcomes of elective labor induction in low-risk term pregnancies

**DOI:** 10.1038/s41598-023-42413-6

**Published:** 2023-09-22

**Authors:** Frida Bengtsson, Cecilia Ekéus, Amelie Hagelroth, Fredrik Ahlsson

**Affiliations:** Department of Women’s and Children’s Health, Uppsala University, Uppsala, Sweden

**Keywords:** Outcomes research, Paediatric research

## Abstract

The rate of labor induction has increased in recent years. The results of previously conducted studies examining associations between elective induction of labor (IOL) and neonatal outcomes have been contradictory. The aim of this study was to examine the intrinsic neonatal risks following IOL. We conducted a population-based cohort study, including all women with recorded low-risk singleton pregnancies at a gestational age between 37 + 0 and 41 + 6 weeks in Sweden from 1999 to 2017. Data were collected from the Swedish Medical Birth register. Two study groups were compared—the elective induction group with the spontaneous labor onset group. The results showed that the rate of elective IOL increased from 7.2% in 1999 to 16.4% in 2017. Elective IOL was associated with a higher OR for chorioamnionitis, bacterial sepsis, intracranial hemorrhage, assisted ventilation, hyperbilirubinemia, APGAR < 7 at 5 min, and neonatal seizures compared to deliveries with spontaneous labor onset. Regarding mortality outcomes, no significant differences were shown between the groups for either early term or full-term deliveries. We conclude that IOL is associated with neonatal complications, although causality could not be established in this observational study. It is important to be aware of the increased risk and perform IOL with caution.

## Introduction

The rate of labor induction is increasing in both Sweden and other high-income countries^1,2^. The most recent statistics published by the Swedish National Board of Health and Welfare in 2019 show that IOL is carried out in 20.7% of Swedish pregnancies, indicating a considerable increase from 7.8% in 1993. However, the proportion of the different indications, medical and elective induction, are not further specified. Inductions are medically indicated if there is a medical reason to why it is advantageous for either the mother or the fetus to interrupt the pregnancy^1^. Elective inductions encompass all non-medically indicated inductions, primarily psychosocial factors. This includes fear of childbirth, previous traumatic birth experience, pregnancy-related pain, risk of unattended delivery (for example, if the pregnant woman lives far away from a hospital and/or has a history of rapid deliveries) or simply that induction is requested by the mother^1^. Internationally it has been suggested that the increased rate of labor induction is owing to an increase in elective inductions, which is supported by the fact that pregnancy-related complications are not rising as fast as the overall rate of induction^3,4^.

Previous studies examining neonatal outcomes of elective IOL have been contradictory. Several studies have presented a decreased neonatal risk among electively induced labors compared to expectant management^5–10^. Associations between elective IOL and a decreased NICU admission^5,9,10^, lower risk of low APGAR-score^10^, a decreased rate of neonatal intubation and respiratory complications^9^ and a decreased rate of meconium-stained amniotic fluid^8^ and meconium aspiration syndrome^10^ has been shown, compared to expectant management. An RCT study presented results showing that elective IOL at 39 weeks was associated with a reduced risk of adverse perinatal outcome (presented as a perinatal composite, defined as one or more of the listed mortality and morbidity outcomes in the study)^11^. It has also been shown that elective IOL is associated with negative neonatal outcomes compared to expectant management^7,12^. For example, increased NICU admission^12^ and a higher rate of hyperbilirubinemia and shoulder dystocia have been presented^7^. Regarding infant mortality outcomes, most of the studies have shown a decreased risk, or at least not an increase, for electively induced labor compared with expectant management^5–7,12^. The rate of perinatal deaths, extended perinatal deaths (including death during the first month after birth), and stillbirths have been shown to decrease with elective induction^5,12^. Accordingly, there is a knowledge gap regarding the impact of elective IOL on neonatal outcome.

Thus, this study aimed to examine neonatal outcomes following elective induction in Sweden. This study will, to our knowledge, be the first Swedish nationwide register study to examine neonatal outcomes of elective labor induction.

## Methods

### Study design

The study was a national population-based cohort study. Data were collected from the Swedish medical birth register (MBR). The diagnoses collected in the register are coded according to the Swedish version of the diagnosis code system International Classification of Diseases, Tenth Revision (ICD-10-SE).

The study included all women with recorded low-risk singleton pregnancies at a gestational age between 37 + 0–41 + 6 weeks at Swedish public hospitals during 1999–2017. The labor onset was spontaneous or electively induced followed by non-instrumental vaginal delivery, operative vaginal delivery, or unplanned caesarian delivery. Both nulliparous and multiparous women were included. Based on the information from MBR one group of electively induced labors and one reference group of deliveries with a spontaneous labor onset were created. The neonatal outcomes were compared between the groups and between different gestational ages (37–38 and 39–41 weeks respectively).

**Figure 1:**
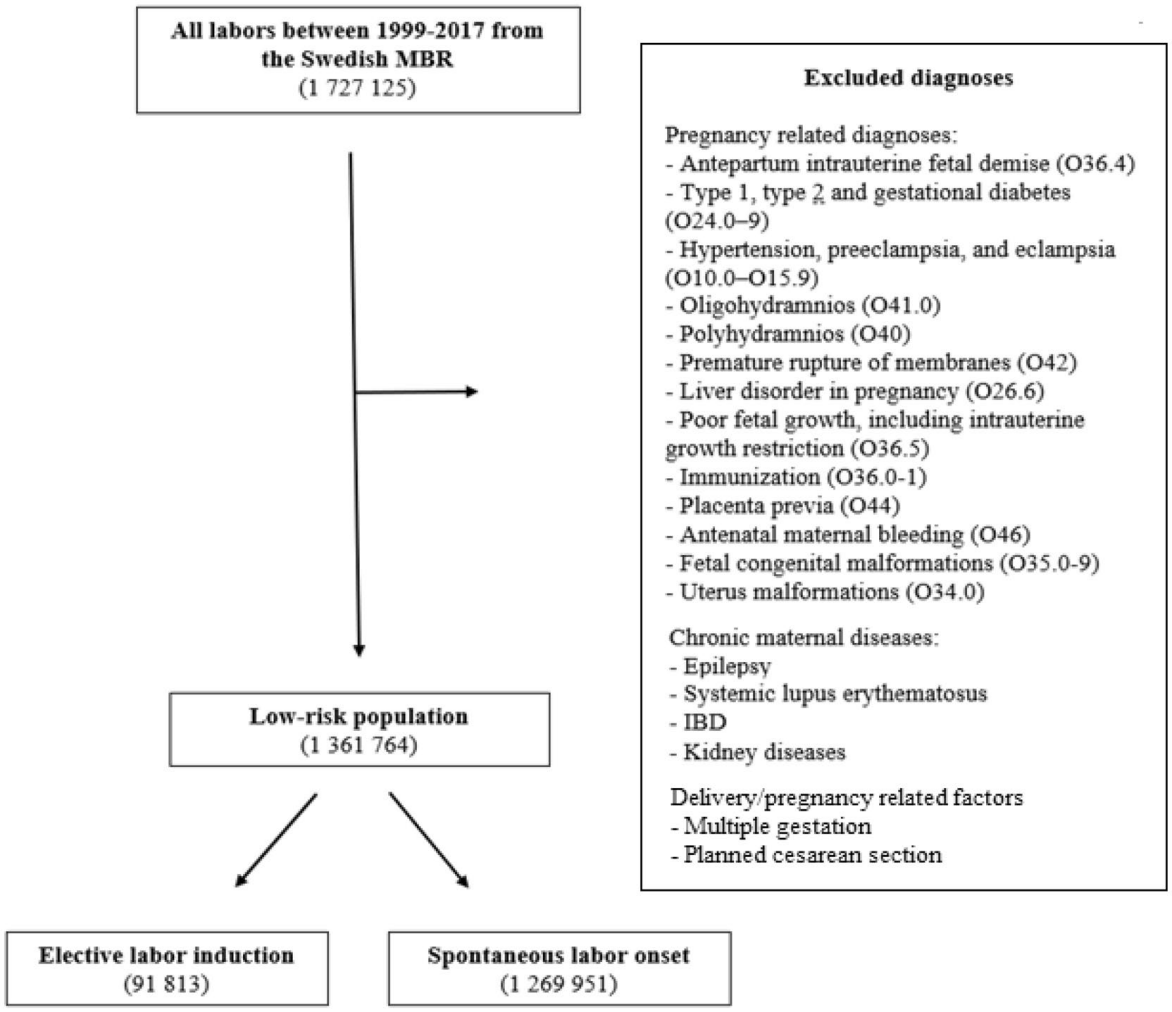
Flowchart describing the process of creating a low-risk population and two separate study groups.

A low-risk population was created by excluding all medical complications that would result in a medical induction. Hence, low risk was defined as the absence of the diagnoses and conditions composing the exclusion criteria. The exclusion criteria, presented in Fig. 1, included pregnancy-related complications (both maternal and fetal conditions that could result in a medically indicated induction) and chronic maternal diagnoses (associated with worse delivery outcomes and therefore routinely requested at antenatal care visits)^13^. Pregnancy-related diagnoses were excluded via ICD-10 diagnosis codes. Information regarding chronic maternal diseases was collected at the first antenatal visit.

### Data analysis

Both maternal, infant, and pregnancy related factors were taken into consideration when defining the study population. The maternal factors included age, BMI, height, parity, and smoking. The infant and pregnancy-related data included gestational age, infant birth weight, ART, and mode of delivery.

Age was categorized into groups of 11–19, 20–29, 30–34, and 35–49 years. BMI was calculated from weight and height collected at the first antenatal care visit and categorized as BMI < 18.5 kg/m^2^ (underweight), 18.5–24.9 kg/m^2^ (normal weight), 25–29.9 kg/m^2^ (overweight), > 30 kg/m^2^ (obesity) or missing. Height was grouped as 130–162 cm, 163–171 cm, and 172–210 cm. Parity was categorized as nulli- or multipara. Information about smoking was collected at the first antenatal care visit and divided into groups of smokers, non-smokers, or missing. Neonatal birth weight was grouped as ≤ 2500 g, 2501–4000 g, and >4000 g. The categorization of gestational age was based on the World Health Organization (WHO) way of dividing term pregnancies into an early-term period, 37–38 weeks, and a late-term period, 39–41 weeks. The delivery mode was categorized as non-instrumental vaginal delivery, operative vaginal delivery, and unplanned caesarian delivery. Assisted reproductive technology (ART) was defined as pregnancies conceived by any type of assisted reproductive technology, such as in vitro fertilization (IVF), oocyte donation (OD), or intracytoplasmic sperm injection (ICSI).

Infant outcomes were divided into mortality and morbidity outcomes. The mortality outcomes included intrapartum fetal death (death of a fetus during labor) and early neonatal death (death of a newborn during the first week). The morbidity outcomes included low APGAR (reported by health care workers via checkboxes on fill in forms), neonatal seizure (P90), intracranial hemorrhage (ICH) (P10 and P52), hypoxic-ischemic encephalopathy (HIE) (P91.6), hyperbilirubinemia (P58.0–5, P58.8–9, P59.0–3, P59.8–9), shoulder dystocia (O66.0), brachial plexus injury (P14), chorioamnionitis (O41.1), bacterial sepsis (P36), assisted ventilation (reported by health care workers via checkboxes on fill in forms) and mode of delivery (reported by health care workers via check boxes on fill in forms). Low APGAR was categorized as APGAR < 7 at 5 min and APGAR ≤ 5 at 10 min. Intracranial hemorrhage included all hemorrhages localized within the intracranial vault. Neonatal seizures were defined as seizures occurring after birth until the end of the neonatal period. Assisted ventilation was defined as mask ventilation > 1 min.

### Statistical analysis

To investigate the associations between electively induced labor and neonatal complications, we used multivariate logistic regression presented as odds ratios (OR) with a 95% confidence interval (CI) using spontaneous labor onset as the reference group.

Two models were used to assess the relationship between the exposure variable elective IOL and neonatal complications, one crude and one adjusted. We adjusted for the following variables: year of birth, maternal age, height, BMI, smoking, infant birthweight, and gestational age. The year of birth was entered as a continuous variable, and all other variables were entered as categories. Distribution of the variables and outcomes were presented as total numbers (N) and rates in percent (%) for the elective induction group and for the reference group, as well as crude and adjusted odds ratio for maternal outcomes in both groups. Data analyzes were performed in statistical program SPSS (SPSS, Statistics for Windows Version 26, IBM Corp; Armonk, NY, USA).

### Ethical approval

The study was approved by the Regional Ethical Review Board in Uppsala on March 16th, 2020 (Dnr: 2020-00113). All research was performed in accordance with relevant national and international guidelines. Informed consent was waived by the Swedish Ethical Review Authority. This was not required as this study was register-based with anonymized data. In general, large registry-based studies in Sweden do not require informed consent. It is explained by Ludvigsson et al. that as long as a registry-based study is deemed ethical by the ethical committee, it is assumed that the participants do not object to the research^14^.

## Results

**Figure 2:**
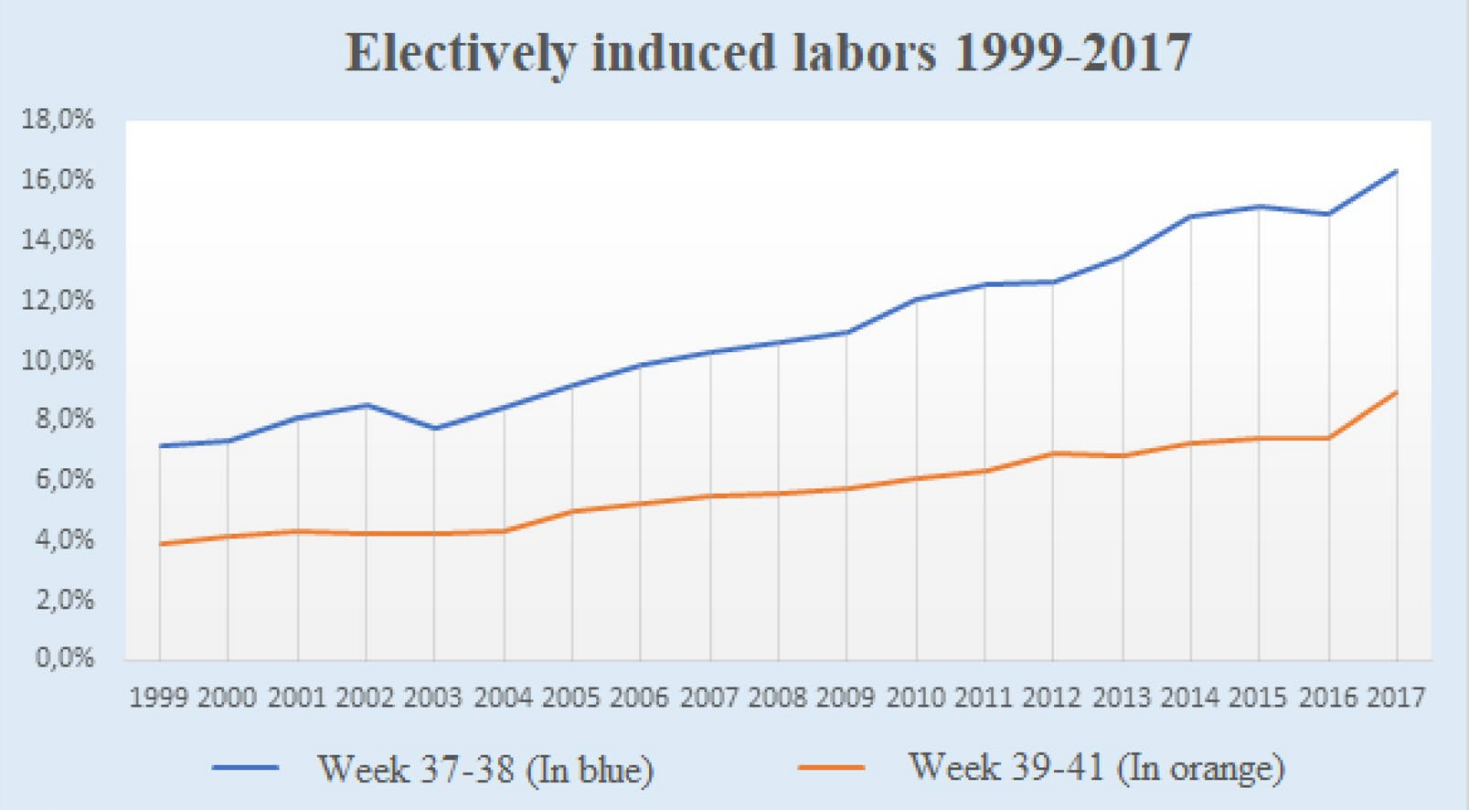
Proportion of low-risk singleton labors with elective induction 1999–2017, at gestational weeks 37–38 and 39–41, respectively.

The study population included 1,361,764 mother infant dyads—91,813 electively induced labors and 1,269,951 labors with spontaneous labor onset. The elective labor induction rate was shown to increase throughout the years (Fig. 2).

### Population characteristics

A summary of the maternal characteristics, fertility treatment, delivery mode, and infant outcome is presented in Table 1. Women who experienced deliveries in which labor was electively induced were more likely to be older (> 35 years), be overweight or obese, be multiparous and smoke, compared to women with spontaneous labor onset. Elective IOL was more commonly carried out in weeks 39–41, than in weeks 37–38, and was associated with a higher prevalence of both operative vaginal delivery and emergency cesarean section. The proportion of emergency cesarean sections was more than twice as high in the elective induction group compared to the comparison group.

**Table 1:** Maternal characteristics, fertility treatment, delivery mode and infant outcome in spontaneous labor and elective induction 1999–2017.

N = 1,361,764	Spontaneous labor onset N = 1,269,951		Elective induction N = 91,813	
	N	(%)	N	(%)
Age (years)				
11–19	20,678	1.6	995	1.1
20–29	579,901	45.7	36,006	39.3
30–34	437,327	34.5	30,758	33.5
35–59	231,059	18.2	23,967	26.1
Height (cm)				
130–162	322,286	26.8	24,295	27.9
163–171	624,588	51.9	44,329	50.8
≥ 172	255,666	21.3	18,565	21.3
BMI (kg/m^2^)				
< 18.5	30,174	2.4	1413	1.5
18.5–24.9	730,641	57.5	43,245	47.1
25–29.9	277,538	21.9	24,233	26.4
≥ 30	114,279	9.0	14,798	16.1
Data missing	117,319	9.2	8124	8.8
Smoking				
Yes	86,865	6.8	7449	8.1
No	1,115,013	87.8	79,168	86.2
Data missing	68,073	5.4	5196	5.7
Parity				
Nulliparous	546,366	43.0	37,532	40.9
Multiparous	723,585	57.0	54,381	59.1
ART pregnancy				
Yes	25,281	2.0	3781	4.1
No	1,244,670	98.0	88,032	95.9
Mode of delivery				
Vaginal	1,110,053	87.4	71,674	78.1
Emergency CS	73,514	5.8	12,620	13.7
VE	86,384	6.8	7519	8.2
Gestational age (weeks)				
37–38	196,859	15.5	24,922	27.1
39–41	1,073,092	84.5	66,891	72.9
Infant birth weight (g)				
< 2500	5913	0.5	603	0.7
2500–4000	1,034,494	81.6	73,628	80.3
> 4000	227,209	17.9	17,483	19.1

### Descriptive outcomes

Elective IOL was shown to be associated with several morbidity outcomes (Table 2). The proportion of all of the examined morbidity outcomes was higher in the elective induction group. The proportion of chorioamnionitis was more than four times higher. Even though the absolute numbers were small, induction of labor was associated with a higher prevalence of the rare outcomes intracranial hemorrhage, HIE and neonatal seizures. The prevalence of mortality did not differ between the groups.

**Table 2:** Descriptive infant outcomes in spontaneous labor and elective induction 1999–2017.

N = 1,361,764	Spontaneous labor onset N = 1,269,951		Elective induction N = 91,813	
	N	(%)	N	(%)
Assisted ventilation	15,294	1.2	1846	2.0
5-min APGAR < 7	8596	0.70*	996	1.1
Sepsis	5824	0.46*	669	0.73*
Chorioamnionitis	1446	0.11*	430	0.47*
Shoulder dystocia	2466	0.19*	239	0.26*
Brachial plexus injury	1950	0.15*	194	0.21*
Seizures	1346	0.11*	131	0.14*
HIE	1292	0.10*	117	0.13*
ICH	362	0.03*	48	0.05*
Early neonatal death	350	0.03*	23	0.03*
Stillborn	46	0.00*	1	0.00*

*All outcomes less than 1% are presented with more precise decimal places.

### Logistic regression data

The outcomes are presented for both deliveries at 37–38 weeks and deliveries at 39–41 weeks, respectively (Table 3). Elective IOL at 37–38 weeks was associated with a significantly increased OR (both crude and adjusted) for chorioamnionitis, bacterial neonatal sepsis, ICH, HIE, APGAR < 7 at 5 min, and assisted ventilation, compared to deliveries with spontaneous labor onset. The highest adjusted ORs were found for chorioamnionitis and intracranial hemorrhage. Elective IOL at 39–41 weeks was associated with a significantly increased OR (both crude and adjusted) for chorioamnionitis, ICH, hyperbilirubinemia, APGAR < 7 at 5 min, neonatal seizures, and assisted ventilation, compared to deliveries with spontaneous labor onset. The highest adjusted OR was found for chorioamnionitis.

**Table 3:** Neonatal outcomes of induced deliveries compared to spontaneous deliveries in 1999–2017.

	Crude OR (95% CI)	P value	Adjusted OR^a^ (95% CI)	P value
**37–38 weeks**				
APGAR score				
< 7 at 5 min	1.40 (1.20–1.62)	< 0.001	1.34 (1.14–1.57)	< 0.001
< 4 at 10 min	0.67 (0.39–1.13)	0.13	0.61 (0.32–1.16)	0.13
Neonatal seizures	1.04 (0.68–1.59)	0.87	1.08 (0.70–1.69)	0.73
Intracranial hemorrhage	2.01 (1.12–3.62)	0.02	2.08 (1.11–3.88)	0.02
HIE	0.79 (0.47–1.32)	0.37	0.76 (0.44–1.33)	0.34
Hyperbilirubinemia	1.63 (1.54–1.73)	< 0.001	1.70 (1.60–1.81)	< 0.001
Shoulder dystocia	1.50 (1.06–2.11)	0.02	1.30 (0.91–1.86)	0.15
Brachial plexus injury	1.32 (0.93–1.89)	0.13	1.17 (0.81–1.70)	0.41
Bacterial sepsis	1.74 (1.46–2.08)	< 0.001	1.45 (1.30–1.62)	< 0.001
Perinatal death	0.71 (0.31–1.63)	0.42	0.88 (0.37–2.07)	0.77
Assisted ventilation	1.45 (1.30–1.62)	< 0.001	1.43 (1.27–1.61)	< 0.001
Chorioamnionitis	3.52 (2.73–4.53)	< 0.001	3.67 (2.80–4.81)	< 0.001
**39–41 weeks**				
APGAR score				
< 7 at 5 min	1.71 (1.59–1.84)	< 0.001	1.45 (1.35–1.57)	< 0.001
< 4 at 10 min	1.11 (0.87–1.41)	0.42	1.24 (0.97–1.60)	0.09
Neonatal seizures	1.48 (1.21–1.80)	< 0.001	1.29 (1.05–1.59)	0.02
Intracranial hemorrhage	1.78 (1.25–2.53)	< 0.001	1.56 (1.08–2.26)	0.02
HIE	1.43 (1.17–1.76)	< 0.001	1.16 (0.93–1.44)	0.19
Hyperbilirubinemia	1.56 (1.48–1.65)	< 0.001	1.43 (1.35–1.52)	< 0.001
Shoulder dystocia	1.42 (1.23–1.64)	< 0.001	1.02 (0.88–1.19)	0.79
Brachial plexus injury	1.47 (1.25–1.73)	< 0.001	1.15 (0.97–1.36)	0.11
Bacterial sepsis	1.62 (1.48–1.78)	< 0.001	1.62 (1.48–1.78)	< 0.001
Perinatal death	0.96 (0.59–1.57)	0.88	0.74 (0.42–1.29)	0.28
Assisted ventilation	1.80 (1.70–1.90)	< 0.001	1.53 (1.45–1.62)	< 0.001
Chorioamnionitis	4.42 (3.92–4.98)	< 0.001	3.19 (2.81–3.62)	< 0.001

*Adjusted for maternal BMI, age, height, smoking, parity, gestational age, year of birth, neonatal birth weight and gender.

When comparing the outcomes of children born at 37–38 weeks to 39–41 weeks, the prevalence of the different outcomes differed between the groups. The adjusted ORs were significantly higher for chorioamnionitis, ICH, and hyperbilirubinemia at 37–38 weeks compared to 39–41 weeks. The adjusted ORs were significantly higher for APGAR < 7 min at 5 min, neonatal seizures, and assisted ventilation at 39–41 weeks compared to 37–38 weeks.

Regarding the mortality outcomes, there were very few cases of intrapartum fetal death and early neonatal death in both the elective induction group and the comparison group. No significant differences were shown between the groups for either early term or full-term deliveries.

## Discussion

Our results showed that the rate of labor induction increased significantly from 1999 to 2017. Since we investigated a low-risk population and excluded factors and diagnoses that would result in medically indicated inductions, this ought to be due to an increase in elective inductions. The reason for the increase can only be speculated upon and several factors are probably part of the explanation. For example, the characteristics of women carrying out labor induction are characteristics that are becoming more and more common among Swedish women in general. According to the results of the present study, women who are older and overweight/obese are more likely to have elective induced labor. The National Board of Health and Welfare in Sweden has presented that the average age of nulliparous women and the prevalence of overweight and obesity at the first antenatal care visit have increased in recent years^15^. These factors might contribute to the increased rate of inductions.

We found that there was a significantly higher OR for both bacterial sepsis and chorioamnionitis in the elective induction group, compared to the spontaneous labor onset group. There are several potential explanations as to why IOL might increase the risk of infections. Firstly, one of the reasons could be the association between labor induction and prolonged labor^4,16^. This is in turn associated with chorioamnionitis^17,18^ and neonatal sepsis^19^. Secondly, there is an association between mechanical induction methods and the risk of infections. Labor induction using an intracervical catheter is associated with an increase in intracervical pathogenic organisms^20^ and performing an amniotomy could result in vaginal micro-organisms reaching the intrauterine milieu^21^.

In addition, the OR for ICH was higher in the elective IOL group compared to spontaneous labor onset. Further, the OR was higher for electively induced labors at 37–38 weeks, compared to electively induced labors at 39–41 weeks. It is well known that ICH is a more common delivery complication among preterm neonates than term neonates, owing to greater maturity of the brain and its vasculature among children born at term. ICH in preterm newborns can occur due to hemodynamic instability, while mechanical factors often are the reason for ICH in term newborns^22^. As mentioned, vacuum extractors are more often needed during induced labors, which can be part of the explanation^1^. It is also possible to assume that full-term infants have a greater brain maturity than early-term infants, even though they are not preterm^22^.

It was also shown that the risk of hyperbilirubinemia was higher in the elective induction group. This might be explained by the fact that induced deliveries often take longer^4,16^ and that vacuum extractor more often are needed for induced labor^1^. A prolonged labor increases the risk of asphyxia, which can cause changes in the liver function^23,24^. Lack of oxygen in the liver increases the risk of hyperbilirubinemia^24^. Regarding vacuum extraction, the procedure can result in a fetal hematoma, which also increases the risk of hyperbilirubinemia due to hemolysis^25^.

When further analyzing mode of delivery, the proportion of emergency cesarean section was higher in the elective induction group. Previously conducted studies have shown an association between emergency cesarean section and negative neonatal outcomes such as higher rate of low APGAR-score, oxygen supply, intubation, low birth weight, and NICU admission, compared to spontaneous vaginal and elective caesarian delivery^26,27^. When interpreting the results, it is important to remember that the higher proportion of cesarean section might be part of the explanation for some of the negative outcomes. It is also important to remember that some of the outcomes possibly overlap. For example, the most frequent cause of neonatal seizures is HIE^28^. Even though a neonate might have a seizure as a result of HIE, both HIE and neonatal seizure may be documented as diagnoses. Several results might overlap this way, which is important to have in mind when interpreting the results.

When comparing our study results to the results of previously conducted studies, and the results of previously conducted studies to each other, it is obvious that the findings are conflicting. A possible explanation might be that the previous studies are largely heterogenous with differences in study design, study population, inclusion- and exclusion criteria, examined outcomes etc. One important difference is that induction method and cervix status are presented in some studies, but not in others. The result of a study only including women with a favorable cervix status will most likely differ from a study only including unfavorable cervix status. Further, comparison groups and contradictive results has previously been widely discussed in several articles^8,10,12^. Previously conducted studies that have presented a comparison group of spontaneous labor onset have been criticized since this does not reflect clinical management. The obstetrician either induces labor or manages the labor expectantly, a spontaneous labor is not a clinical choice^8,10,12^. The comparison group of this register study is therefore an important limitation to bring up. The most accurate way to compare the two alternatives would be to perform RCTs. Further, one major difference between the present study and other studies in this field, which makes it hard to compare the results, is that our objective differs from the objectives of previously conducted studies. The objective of the present study is to examine if elective induction is associated with risks for the neonate. The single most important factor to focus on in order to end up with reliable results, is to only include low-risk women in the study population, since the reason for inducing in risk pregnancy is to improve birth outcomes. To our knowledge, none of the previously conducted studies has excluded as many diagnoses and conditions as we have in this study. Apart from differences regarding the structure of the study there also are more general differences between the countries in which the studies are conducted, for example, demographic differences and differences in labor care.

A limitation to the study was that the Swedish MBR did not include information regarding bishop score, induction method, CTG parameters, lactate, and pH values. Further, the register lacks information regarding the indication of the inductions, whether they are medically or electively indicated. In this article elective induction therefore was defined as IOL without recorded conditions that could result in medical induction. Thus, several diagnoses and conditions were excluded, both maternal and fetal. This raise the question whether the absence of recorded reasons is similar to actually having no medical reason for induction. It is an important limitation to point out, however, to our knowledge it is the most appropriate way to examine elective induction in a register study based on diagnosis codes. It should also be mentioned that it is clinically difficult to label IOL to medical or non-medical since the decision often is made balancing risk and benefit. It is also important to remember that some of these labors might be induced in order to prevent conditions that are likely to appear later on, such as macrosomia, shoulder dystocia etcetera (especially since our results indicate that over a quarter of the elective inductions are performed before full-term). It would be hard to argue that these inductions would be non-medical, however, it is not possible to exclude them from the study population since there is no recorded diagnosis code. When creating a study population based largely on diagnose codes, it is also important to remember that the diagnose system itself can be a source of error. In addition, information about chronic diseases and maternal use of tobacco was self-reported, which affects the reliability of the data. Further, the register did not include reliable information regarding socioeconomic status, thus this is not adjusted for. Another limitation to this study is that it has not been presented how induction differs over time. Population characteristics, such as obesity, has changed throughout the years 1999–2017^15^, which could result in a higher amount of inductions being performed^29^. However, we have taken this into consideration by adjusting for several factors such as BMI and birth year. Lastly, the study only examined short-term outcomes.

The major strength of the present study is the large study population, collected from the Swedish MBR. This enables rare outcomes to be examined. Even though the register lacks some information, mentioned in “limitations”, it is the most suitable register and source of information for the present study^1,30,31^. As mentioned, the only criterion that needs to be fulfilled in order to be included in the register is that the pregnancy resulted in a delivery. This, together with the fact that the inclusion and exclusion criteria only included diagnosis codes, contributed to a low risk of selection bias. Moreover, we did not have to select study participants from exposure status and we did not have to deal with losses to follow-up, which could have generated bias related problems. Another advantage of performing a Swedish register study is that 99% of Swedish childbirths take place in public hospitals^1,31^ and that information regarding 97–99% of the Swedish deliveries are included in the Swedish MBR^32^. A quality study of the MBR has been conducted and presented that the register is suitable for handling hard data, but that the evaluation of other types of data should be handled with care^33^. Lastly, as mentioned above, a major advantage is that the objective of the study differs from the objectives of previously conducted studies. To our knowledge, this is the first study that aimed to examine the intrinsic risks of labor induction.

The present study has contributed to the state of research by presenting risks associated with elective labor induction in Sweden. This could be a step toward a change of the clinical practice—a clinical practice that needs to be reviewed and updated in order to equalize the health care across the country. Based on the result of the present study showing an association between IOL and chorioamnionitis as well as neonatal sepsis, preventive treatment with prophylactic antibiotics for women undergoing IOL could be a suggestion for a future change in the clinical practice. However, further research, especially RCT studies, are needed before such recommendation could be made.

## Conclusion

The present study showed that elective IOL has increased from 1999 to 2017 and that electively induced labors are associated with negative infant outcomes compared to labors with a spontaneous labor onset. Consequently, elective IOL should be performed with caution and the criteria of elective induction should be continuously reviewed as research is advancing. Pregnant women should always receive information about labor induction, advantages and disadvantages, in order to make an informed decision. The findings of the study are presented as associations and not correlations. Further research is needed, both for short- and long-term outcomes and in order to examine strategies for risk prevention in the future.

## Data Availability

The data that support the findings of this study are available from the Swedish Medical Birth Register, held by the National Board of Health and Welfare. Restrictions apply to the availability of these data, which were used under license for the current study, and so are not publicly available. Data are however available from one of the authors (CE) upon reasonable request and with permission of the National Board of Health and Welfare.
